# Supplementary material for: Contextualizing gender disparities in online teaching evaluations for professors
Source: PLoS One. 2023 Mar 16;18(3):e0282704. doi: 10.1371/journal.pone.0282704 (PMC10019737; doi:10.1371/journal.pone.0282704)
Supplement: S1 Table — (DOCX) [file pone.0282704.s001.docx]

**S1 Table. Top 10 departments in each field.**

| **Field** | **# of professors** | **Field** | **# of professors** |
| --- | --- | --- | --- |
| ***Applied Sciences*** |  | ***Natural Sciences*** |  |
| Business | 27529 | Biology | 33176 |
| Accounting | 14009 | Chemistry | 20298 |
| Management | 7434 | Science | 12534 |
| Marketing | 6362 | Physics | 10049 |
| Finance | 4741 | Geology | 3832 |
| Architecture | 3008 | Biological Sciences | 1355 |
| Agriculture | 1634 | Astronomy | 652 |
| Hospitality | 1507 | Earth Science | 507 |
| Business Administration | 1285 | Environmental Science | 478 |
| Counselling | 904 | Biochemistry | 367 |
| ***Math & Computing*** |  | ***Engineering*** |  |
| Mathematics | 62531 | Engineering | 16032 |
| Computer Science | 23517 | Electrical Engineering | 1327 |
| Information Science | 1934 | Mechanical Engineering | 1265 |
| Statistics | 1383 | Civil Engineering | 779 |
| Computer Information Systems | 979 | Chemical Engineering | 471 |
| Information Technology | 770 | Aviation | 384 |
| Information Systems | 520 | Automotive Technology | 260 |
| Technology | 398 | Industrial Engineering | 220 |
| Library Science | 363 | Engineering Technology | 145 |
| Computer Engineering | 267 | Aviation Pilot | 143 |
| ***Medicine Health*** |  | ***Social Sciences*** |  |
| Nursing | 12262 | Psychology | 38036 |
| Health Science | 10327 | Communication | 24110 |
| Physical Education | 7810 | Economics | 16839 |
| Medicine | 1740 | Political Science | 16761 |
| Kinesiology | 1404 | Sociology | 16287 |
| Nutrition | 620 | Criminal Justice | 8861 |
| Human Development | 541 | Law | 8643 |
| Health | 381 | Anthropology | 8204 |
| Health & Physical Education | 346 | Geography | 5813 |
| Exercise & Sport Science | 345 | Social Science | 5178 |
| ***Education*** |  | ***Humanities*** |  |
| Education | 23874 | English | 81393 |
| Child Development | 755 | History | 29633 |
| Early Childhood Education | 437 | Languages | 20213 |
| Special Education | 288 | Music | 17044 |
| Freshman Seminar | 206 | Philosophy | 14836 |
| Academic Services | 199 | Fine Arts | 11990 |
| Student Development | 189 | Theater | 6840 |
| College Success | 174 | Humanities | 6796 |
| Elementary Education | 154 | Religion | 5418 |
| Curriculum & Instruction | 152 | Art History | 5311 |
