# Supplementary material for: Contextualizing gender disparities in online teaching evaluations for professors
Source: PLoS One. 2023 Mar 16;18(3):e0282704. doi: 10.1371/journal.pone.0282704 (PMC10019737; doi:10.1371/journal.pone.0282704)
Supplement: S2 Table — Representative sentences have been replaced with gender-neutral pronouns. (A) five-star reviews. (B) one-star reviews. (DOCX) [file pone.0282704.s002.docx]

**S2 Table. Topics in five- and one-star reviews and statistics of their probabilities.** Representative sentences have been replaced with gender-neutral pronouns. (A) five-star reviews. (B) one-star reviews.

**(A)** **five-star reviews**

| **Topic** | **Mean** | **95% CI** | **Representative sentence** |
| --- | --- | --- | --- |
| **Overall** |  |  |  |
| chance; regret; highly recommend | 0.009 | [0.009, 0.009] | I feel bad for anyone that doesn't get to take them. |
| class; student; class fun | 0.003 | [0.003, 0.003] | I wish there were more classes I can take that they teaches. |
| instructor; good instructor; great instructor | 0.004 | [0.004, 0.004] | Fantastic instructor, pushes, encourages, and supports students to push past their fears and self imposed boundaries. |
| lecturer; great lecturer; good lecturer | 0.01 | [0.01, 0.01] | They's a great lecturer, and even though there's a lot of reading, you can easily figure out what's important through their lecture hints. |
| professor; good professor; professor great | 0.011 | [0.011, 0.011] | I found Professor Person to be fair, intelligent, and very skilled in their subject. |
| teacher; good teacher; teacher great | 0.003 | [0.003, 0.003] | Person really cares about their students and wants everyone to learn and to think. |
| woman; lady; teacher | 0.011 | [0.011, 0.011] | This lady takes their time if you don’t understand they will make sure you do by the end of the class! |
| **Teaching** |  |  |  |
| candy; pizza; snack | 0.002 | [0.002, 0.002] | Our class was small and they invited us over for dinner at the end of the semester. |
| feedback; give feedback; feedback work | 0.002 | [0.002, 0.002] | Just follow their feedback and you'll do awesome. |
| learn lot; fun learn; work learn | 0.007 | [0.007, 0.007] | I actually came away having learned a lot. |
| lecture; lecture interesting; class lecture | 0.002 | [0.002, 0.002] | Lectures were mostly interesting, and were interactive with the i-clicker. |
| material; understand material; explain thing | 0.002 | [0.002, 0.002] | They never got impatient or irritated, and did not mind going over topics you may have been unclear about. |
| office hour; helpful office; help office | 0.001 | [0.001, 0.002] | I once sat with them in office hours for an hour to just talk and I never go to office hours! |
| personal story; story interesting; story great | 0.002 | [0.002, 0.002] | I got to go to dinner with them, they was really invested in sharing their story and offering me advice. |
| respond email; email quickly; answer email | 0.002 | [0.002, 0.002] | They is also very quick to respond by email if you have any questions, and is very flexible and happy to help you workout any issues. |
| speech; public speaking; speaker | 0.003 | [0.003, 0.003] | You video tape yourself for the class is online so it may help with learning to adapt to public speaking anxiety. |
| willing help; want succeed; extra help | 0.002 | [0.002, 0.002] | If you need help they's more than willing to help you with whatever is needed. |
| writing; writing class; improve writing | 0.001 | [0.001, 0.001] | This class was much more work then expected, but showed me that I could be a pretty good writer. |
| **Personal** |  |  |  |
| accent; thick; british | 0.003 | [0.003, 0.003] | The beginning is a little rough because they has a thick Scottish accent but you get used to it. |
| hot; attractive; sexy | 0.004 | [0.003, 0.004] | They is young, super smart and YES we all want to look/dress like them when we group up! |
| humor; sense humor; intelligent | 0.006 | [0.005, 0.006] | I think the reason many people don't get Person' sense of humor is the generation gap, to be honest. |
| smart; intelligent; caring | 0.003 | [0.003, 0.003] | Very witty and has a lot of great one liners. |
| **Material** |  |  |  |
| class lab; lab class; lab easy | 0.003 | [0.003, 0.003] | Every lab has clear detailed instructions to follow and participate with your partner. |
| note; class note; note study | 0.007 | [0.006, 0.007] | uses overheads and writes the notes for you which you then transcribe on your note pad. |
| reading; lot reading; reading class | 0.011 | [0.011, 0.011] | The works they chooses for reading are very interesting and they clearly goes through the material to make it as easy as possible to understand. |
| slide; powerpoint; study powerpoint | 0.006 | [0.006, 0.006] | Make sure you printout their powerpoint slideshow before class, that way note taking is so much easier and it makes studying a breeze. |
| study guide; guide test; test study | 0.004 | [0.004, 0.004] | They gives study guides for their tests and 2 easy papers. |
| syllabus; syllabus clear; follow syllabus | 0.009 | [0.009, 0.009] | They made a "road map" overview of the course and stuck to it and the syllabus, so you knew exactly what you were going to do that day. |
| textbook; buy book; book class | 0.003 | [0.003, 0.003] | No textbook, you only have to buy the 12 dollar song book packet. |
| **Structure** |  |  |  |
| work; lot work; work hard | 0.007 | [0.007, 0.007] | Show up and do quality work and you will be fine. |
| work; study; effort | 0.008 | [0.008, 0.008] | No way you shouldn't get an A! |
| **Evaluation** |  |  |  |
| attendance; attendance mandatory; class attendance | 0.176 | [0.176, 0.176] | Attendance is mandatory, but the class is so much fun that you won't have a problem going. |
| exam; test hard; exam easy | 0.099 | [0.099, 0.099] | Although their examinations are difficult I would say that I took away the most knowledge from their course relative to other ACC courses. |
| group project; project class; project fun | 0.067 | [0.067, 0.067] | The projects are straight forward and the final project is fun. |
| homework; lot homework; class homework | 0.017 | [0.017, 0.017] | The homework they gives help you to learn and retain information because you have to remember what you have learned to build off of. |
| lecture; lecture test; lecture note | 0.014 | [0.014, 0.014] | Their tests were primarily based on what was said in lecture, but my favorite thing about them was meeting after class over a snack to go through the readings for all interpretations. |
| midterm final; midterm easy; guide midterm | 0.014 | [0.014, 0.014] | The midterm is a little difficult because it is all vocab however they gives you a study guide and they does their best to prepare you for it. |
| quiz week; quiz test; quiz class | 0.013 | [0.013, 0.013] | Quizzes are a little redundant and the computers are a little slow but all and all worthwhile. |
| research paper; write essay; write paper | 0.015 | [0.015, 0.015] | If you plan ahead, you really don't have to write a final paper. |
| test review; review sheet; review exam | 0.015 | [0.015, 0.015] | They does a test review the class meeting before the test which helps a lot. |
| **Grading** |  |  |  |
| credit; extra credit; credit opportunity | 0.022 | [0.022, 0.022] | They ALWAYS gives credit and recognition when it's due. |
| grade paper; grader paper; grading | 0.017 | [0.017, 0.017] | Your grade is mostly composed of the papers but they's not a super hard grader. |
| grading; fair grader; easy grader | 0.023 | [0.023, 0.023] | They is a tough grader but getting a good grade is pretty easy. |
| **Subject** |  |  |  |
| accounting; financial; major | 0.003 | [0.003, 0.003] | They was annoyed by my lack of attends(they's really on top of it) but I appreciated their teaching method with accounting. |
| acting; theater; watch movie | 0.002 | [0.002, 0.002] | I could not have asked for a better acting instructor and I cannot wait to take a class from them again. |
| art; art history; art class | 0.002 | [0.002, 0.002] | their classes are tough for some people because if you don’t spend a lot of time outside art classes you would never get good work. |
| bible; religion class; religious | 0.003 | [0.003, 0.003] | Very entertaining and funny in class, really gives a different perspective on the bible than most people are used to. |
| biology; biology class; biology major | 0.006 | [0.006, 0.006] | It's very technical and it's easy to be lost unless you are into biology software. |
| calculus; calculus class; trigonometry | 0.004 | [0.004, 0.004] | funny stories and advice about life and they actually makes calc pretty easy. |
| chemistry; organic; lab | 0.002 | [0.002, 0.002] | They was the one who inspired me to be a chemistry major! |
| clinical; medical; medical school | 0.006 | [0.006, 0.006] | I really enjoyed all of their lectures and this class is preparing me for my future in medicine. |
| economics; economic; economics class | 0.006 | [0.006, 0.006] | Whenever I take an economics course, I want it to be taught by Person. |
| english class; english professor; english teacher | 0.001 | [0.001, 0.002] | Overall great teacher, and I would recommend anyone with basic knowledge of English to take their reading course. |
| french; france; language | 0.003 | [0.003, 0.003] | took this intermediate French class over the summer M-F 10 am, but I genuinely enjoyed their teaching style which made me want to come to class. |
| history class; history teacher; history professor | 0.003 | [0.003, 0.003] | I have always done poorly in previous history classes and thanks to them I am interested in taking more history classes. |
| law; criminal; criminal justice | 0.006 | [0.006, 0.006] | They is the only person that can make Contract Law @ 9 am anywhere close to interesting. |
| literature; literature class; literary | 0.002 | [0.002, 0.003] | They provokes thought on the literature and gets you to understand what it means. |
| math; math class; math teacher | 0.007 | [0.007, 0.007] | Professor Person actually helped me become a better math student! |
| music; piano; musician | 0.002 | [0.002, 0.002] | I didn't know anything about music before taking their class but I can play popeye the sailor man on the flute so i've progressed a lot. |
| nursing; nurse; instructor | 0.002 | [0.002, 0.002] | They is tough but you will become a better nurse if you follow their guidelines. |
| philosophy; philosophy class; philosopher | 0.001 | [0.001, 0.001] | Very passionate about philosophy and will do their best to accommodate you if something comes up. |
| physics; physics class; professor | 0.006 | [0.006, 0.006] | You won't be disappointed taking their class if you want to learn something about fluid dynamics. |
| political; politics; political science | 0.002 | [0.002, 0.002] | They's even done optional lectures to give political career advice and helped me score a high level job in DC. |
| programming; computer science; java | 0.002 | [0.002, 0.002] | I started this class with almost no computer knowledge at all and I left with an A and I knew how to use the computer. |
| psychology; psychology class; psychology major | 0.01 | [0.01, 0.01] | They also gives bonus for participating in psych research, writing papers, or attending class (sometimes). |
| science class; science major; class science | 0.002 | [0.002, 0.002] | Which is difficult to do in a science class for nonscience majors. |
| sociology; social; social work | 0.002 | [0.002, 0.002] | Person really connects with people and has passion for Sociology. |
| spanish; latin; speak | 0.001 | [0.001, 0.001] | I took their Spanish 100 class and passed with an A, now I will be taking their Spanish 101 class next semester! |
| statistics; statistics class; good statistics | 0.003 | [0.003, 0.003] | They is extremely enthusiastic about statistics, and makes sure that everyone understands EVERYTHING. |
| workout; exercise; fitness | 0.011 | [0.011, 0.011] | Your physical type doesn't matter as long as you stick to the workout and show up to class. |
| **Other/not specified** |  |  |  |
| semester; taught; wish taught | 0.004 | [0.004, 0.004] | But who am I to say? |
| strongly recommend; recommend friend; definitely recommend | 0.009 | [0.009, 0.009] | I would recommend to everyone. |
| ta good; ta helpful; ta great | 0.007 | [0.007, 0.007] | Their tests are not too bad but sometimes their TAs grade a bit harshly. |

**(B)** **one-star reviews**

| **Topic** | **Mean** | **95% CI** | **Representative sentence** |
| --- | --- | --- | --- |
| **Overall** |  |  |  |
| avoid; avoid cost; avoid possible | 0.031 | [0.031, 0.031] | Be cautious before taking any class with them. |
| bad instructor; horrible instructor; terrible instructor | 0.004 | [0.004, 0.004] | Terrible instructor and makes their students feel poor. |
| bad professor; horrible professor; terrible professor | 0.065 | [0.065, 0.066] | This was the worst prof I ever had. |
| bad teacher; horrible teacher; terrible teacher | 0.08 | [0.079, 0.08] | They is just a terrible teacher and unbelievably paranoid. |
| bad; horrible; terrible | 0.006 | [0.006, 0.006] | Simply put: they's dreadful. |
| change major; major class; switch major | 0.005 | [0.005, 0.006] | they makes me want to change my major. |
| class bad; life bad; career bad | 0.008 | [0.008, 0.008] | This is the worst class that I have ever taken! |
| class woman; teacher; class lady | 0.003 | [0.003, 0.003] | The way this woman treats their students is unreal. |
| drop class; student drop; drop half | 0.014 | [0.014, 0.014] | I can see why students are dropping the class and switching. |
| fire; head department; department chair | 0.005 | [0.005, 0.005] | Administration needs to get rid of this guy. |
| hate class; dread class; class horrible | 0.01 | [0.01, 0.01] | Every aspect of Person's class was garbage. |
| lecturer; bad lecturer; terrible lecturer | 0.002 | [0.002, 0.002] | They is a poor lecturer. |
| retire; need retire; retire year | 0.004 | [0.004, 0.004] | They is so elderly that they needs to retire. |
| review; rating; good rating | 0.002 | [0.002, 0.002] | The negative reviews are true. |
| tutor; tutoring center; help tutor | 0.005 | [0.005, 0.005] | I advise you seek tutoring help and say goodbye to a normal life once you walk in their class. |
| waste; waste time; waste money | 0.006 | [0.006, 0.006] | If you're looking to waste your time. |
| **Teaching** |  |  |  |
| answer question; explain material; explain thing | 0.024 | [0.024, 0.025] | Did not know the material during presentation, let alone well enough to answer questions. |
| asleep class; fall asleep; awake class | 0.004 | [0.004, 0.004] | Ten redbull won't keep you awake in their class, i know a moron when i see one. |
| care student; rude student; student feel | 0.025 | [0.025, 0.025] | They needs to not get mad when students ask their questions sometimes. |
| class talk; class time; spend class | 0.006 | [0.006, 0.006] | They spent a half hour talking about washing machines and totally irrelevant stuff! |
| feedback; give feedback; feedback work | 0.003 | [0.003, 0.003] | Also, do not expect any feedback for your work. |
| help helpful; extra help; bother help | 0.007 | [0.007, 0.007] | They did not help when I went for help. |
| late class; minute late; miss class | 0.005 | [0.005, 0.005] | First, they was 15 to 30 min late almost every class. |
| learn thing; teach learn; teach material | 0.014 | [0.014, 0.014] | My only concern is that you don't learn anything. |
| lecture boring; class boring; lecture long | 0.021 | [0.021, 0.021] | I can never hear their lectures through their mumbling and rely mainly on youtube to teach me. |
| office; office hour; help office | 0.003 | [0.003, 0.003] | However, one time at office hours they was moderately encouraging. |
| problem board; write board; board explain | 0.003 | [0.003, 0.003] | Writes on the board and is already talking about the next thing while you're struggling to keep up with them. |
| respond email; answer email; reply email | 0.009 | [0.009, 0.009] | THEY NEVER REPLIES ANY EMAILS and they is IMPOSSIBLE to get a hold of. |
| **Personal** |  |  |  |
| accent; thick accent; heavy accent | 0.004 | [0.004, 0.004] | Do not take their course, they is a hard marker and they has a strong a non-English accent. |
| boring; dull boring; boring extremely | 0.006 | [0.006, 0.007] | Its tedious, boring, pointless. |
| favorite; play favorite; pick favorite | 0.003 | [0.003, 0.003] | they was an idiot who played favorites. |
| humor; sense humor; funny | 0.004 | [0.004, 0.004] | They definitely tries way too hard to be funny. |
| lady; rude; woman horrible | 0.006 | [0.006, 0.006] | There are no words to describe this woman other than evil. |
| nursing; doctor; professor | 0.003 | [0.003, 0.003] | Dr. Person is disrespectful to students and often times would make mistakes on their own examples. |
| opinionated; opinion wrong; argue | 0.005 | [0.005, 0.005] | If you don't agree with their you are wrong. |
| rude; condescending; arrogant | 0.009 | [0.009, 0.009] | They is very rude and talk about attitude. |
| voice; mumble; talk fast | 0.012 | [0.012, 0.012] | Plus they talks slower than anyone on the face of the planet. |
| **Material** |  |  |  |
| class note; write note; page note | 0.006 | [0.006, 0.006] | Taking notes is so important and they cares about participation, class average is 60-70! |
| lab report; lab class; lab manual | 0.002 | [0.002, 0.002] | Their labs are taking screenshots and putting them in your VEE interface. |
| read chapter; chapter class; lot reading | 0.007 | [0.007, 0.007] | always ended class early, never read chapters in class. |
| read powerpoint; read slide; powerpoint presentation | 0.01 | [0.01, 0.01] | They says they are based on the slides but nope! |
| study guide; guide test; test study | 0.004 | [0.004, 0.004] | It's impossible to tell what's important, their study guides aren't helpful, and the final exam covers stuff they never even mentioned. |
| syllabus; follow syllabus; change syllabus | 0.003 | [0.003, 0.003] | They makes the syllabus very unclear, they doesn't have many helpful hints. |
| text book; book class; book test | 0.02 | [0.02, 0.02] | Very unclear and unhelpful - had to teach myself through the textbook. |
| **Structure** |  |  |  |
| easy class; level class; hard class | 0.016 | [0.016, 0.016] | This class is extremely harder than it needs to be. |
| instruction; clear instruction; instruction unclear | 0.003 | [0.003, 0.003] | Their "instructions" are lengthy and ambiguous. |
| online class; online course; teach online | 0.005 | [0.005, 0.005] | Tbh take this class online because it is sort of a waste of time otherwise. |
| work; busy work; work load | 0.007 | [0.007, 0.007] | too much busy work and very unhelpful and arrogant personality! |
| **Evaluation** |  |  |  |
| assignment; give homework; assignment due | 0.016 | [0.016, 0.016] | They assigns a huge load of homeworks from mastering physics. |
| attendance mandatory; class attendance; attendance policy | 0.004 | [0.004, 0.004] | Attendance is not mandatory but you need to attend in order to pass. |
| exam; test hard; test difficult | 0.025 | [0.025, 0.026] | Their tests are extremely hard like hell. |
| group project; group work; project class | 0.002 | [0.002, 0.002] | They is hard to reach and gives you no response or feedback regarding projects. |
| lecture test; lecture exam; class lecture | 0.004 | [0.004, 0.004] | The lectures aren't helpful nor recorded by them and the exams contain material that was not covered prior to testing. |
| midterm final; class midterm; midterm exam | 0.003 | [0.003, 0.003] | We handed in 4 cases prior to the midterm and had zero marks going into it. |
| page paper; write essay; grade essay | 0.009 | [0.009, 0.009] | Failed every one of my papers saying they didn't follow their guidelines. |
| quiz test; pop quiz; quiz class | 0.005 | [0.005, 0.006] | I heard their say that they took delight in making quizzes extra hard. |
| test review; review sheet; review exam | 0.003 | [0.003, 0.003] | They does not review what is going to be on a test prior to the test. |
| **Grading** |  |  |  |
| care pass; pass fail; want fail | 0.006 | [0.006, 0.006] | Too hard, and I utterly failed. |
| class average; average test; average exam | 0.003 | [0.003, 0.003] | The class averages for each test was incredibly low. |
| credit; extra credit; partial credit | 0.004 | [0.004, 0.004] | Mr. Person don't give out extra credit be prepare for a long semester. |
| curve test; curve class; grade curve | 0.003 | [0.003, 0.003] | Does grade on a curve, but not worth an easy A! |
| gpa; gpa class; ruin gpa | 0.003 | [0.003, 0.003] | if you want a lower gpa then you can take their class . |
| grade paper; grading; bad grade | 0.004 | [0.004, 0.004] | very hard, crazy, even if u try very hard for a paper the best grade u will earn is low B. don’t take them. |
| grade test; grade exam; grader test | 0.003 | [0.003, 0.004] | You don't know if you failed or aced a test until you get the grade back. |
| grading; grade class; grading system | 0.035 | [0.034, 0.035] | but one good thing is that they def doesn’t look at numbers when giving grades. |
| marker; hard marker; tough marker | 0.002 | [0.002, 0.002] | I thought they was an extremely tough marker. |
| take point; deduct point; lose point | 0.002 | [0.002, 0.002] | Takes 4 points off of one question if you don't circle your answer! |
| **Subject** |  |  |  |
| accounting; accounting class; cpa | 0.001 | [0.001, 0.001] | i should've listened to the people who said not to take this guy especially accounting online. |
| biology; biology class; biology major | 0.002 | [0.002, 0.002] | Bio for this prof is in the dictionary under Dbag. |
| calculus; calculus class; precalculus | 0.003 | [0.003, 0.003] | Just go to lecture and sign the attendance sheet, then leave to the library to teach yourself calculus. |
| chemistry; chemistry class; organic | 0.003 | [0.003, 0.003] | this could be due to the fact that they is a researcher not a teacher and never taught chem 105. |
| drawing; art class; art history | 0.002 | [0.002, 0.002] | They taught to their opinion of what was good in art, and was uneven on assignments. |
| economics; economics class; economics major | 0.001 | [0.001, 0.001] | I expected economics to be difficult, but Person's poor instruction has made it a very dismal course for me. |
| history class; history major; class history | 0.002 | [0.002, 0.002] | i would not recommend taking a class with them, they grades way to hard for anyone without a history degree. |
| law school; lawyer; criminal justice | 0.004 | [0.004, 0.004] | Unless you are a law nerd who enjoys entertaining hypotheticals which have no application to the real world, get out. |
| math class; math teacher; math professor | 0.007 | [0.007, 0.007] | Do not take their class if math is not your best subject. |
| music; music major; music class | 0.002 | [0.002, 0.002] | All I learned was that they expected us to tell the difference between types of saxophone! |
| physics; physics class; high school | 0.002 | [0.002, 0.002] | Unless you can learn physics based on theory alone! |
| political; political science; religion class | 0.003 | [0.003, 0.003] | unless you already know everything there is to know about religion don’t take their class. |
| programming; computer science; coding | 0.003 | [0.003, 0.003] | This teacher really needs to learn more about computers before they teaches about them! |
| psychology; psychology class; psychology major | 0.001 | [0.001, 0.001] | Person's class is completely off-putting for anyone interested in psychology. |
| spanish; speak; fluent | 0.002 | [0.002, 0.002] | Spanish was my major, but I'm changing it because I don't want to have them as a teacher ever again. |
| speak english; english major; language barrier | 0.006 | [0.006, 0.006] | Not to mention they doesn't know how to speak it all or knows grammar. |
| speech; public speaking; speech class | 0.002 | [0.002, 0.002] | I felt like I spent the entire semester writing speeches and their grading didn’t reflect your performance. |
| statistics; statistics class; teach statistics | 0.001 | [0.001, 0.001] | Apparently they thinks that stats in an English course. |
| theater; acting; watch movie | 0.002 | [0.002, 0.002] | horrible and expect you to know everything about the movies like you took a film class before. |
| writing; writing class; writing center | 0.003 | [0.003, 0.003] | your hands burn by the end of class cause you’ve been writing for an hour and 15 minutes straight. |
| **Other/not specified** |  |  |  |
| class ta; ta grade; ta teach | 0.003 | [0.003, 0.003] | My TA was a God send and I'm only passing the class because of them. |
| positive; negative; written | 0.003 | [0.003, 0.003] | and it is not. |
